# Spinal Anesthesia in a Patient With Cold Agglutinin Disease Presenting for Total Knee Arthroplasty in a Community Hospital Setting

**DOI:** 10.7759/cureus.80825

**Published:** 2025-03-19

**Authors:** Cameron Thiele, Aamil Patel, Rebecca L Johnson

**Affiliations:** 1 Department of Anesthesiology and Perioperative Medicine, Mayo Clinic, Rochester, USA

**Keywords:** autoimmune hemolytic anemia (aiha), cold agglutinin disease, intraoperative hypothermia, spinal anesthesia, thermoregulation

## Abstract

Idiopathic cold agglutinin disease is a form of autoimmune hemolytic anemia (AIHA) characterized by autoantibody-mediated red blood cell (RBC) agglutination and hemolytic anemia at colder temperatures. Due to the increased risk of clinical manifestations of this condition at cold temperatures, this condition presents unique thermoregulatory considerations perioperatively. There is a risk of hypothermia in patients who undergo both general and regional anesthesia due to impairments in thermoregulatory control. However, there is a paucity of literature detailing perioperative considerations for this patient population who undergo neuraxial anesthesia. In this report, the physiology of perioperative hypothermia and the thermoregulatory impairments seen in both general and neuraxial anesthesia are reviewed. This case report details the perioperative management and warming strategies for an 83-year-old female with a history of idiopathic cold agglutinin disease who underwent spinal anesthesia for an elective primary total knee arthroplasty (TKA) in a community hospital setting. This report demonstrates that under a strict temperature management strategy, either spinal or general anesthesia may be considered for this patient population. Pre-operative optimization, stability of symptoms, the frequency of laboratory monitoring required, and the need for rapid cold agglutinin-directed therapy influence the decision as to whether these cases can be safely performed in an ambulatory community hospital setting versus a tertiary care center.

## Introduction

Autoimmune hemolytic anemias (AIHAs) consist of warm, cold, or mixed autoantibodies directed against the red blood cell (RBC) membrane. These autoantibodies may be idiopathic (primary) or related to an underlying condition such as trauma, infection, or autoimmune disease (secondary) [1]. Cold agglutinin disease, a form of AIHA, accounts for 15% of AIHA cases. This condition is predominantly an IgM autoantibody-mediated process that activates complement-driven extravascular hemolysis at cooler temperatures, such as in the peripheral circulation [1,2]. Clinical manifestations include cold-induced circulatory symptoms related to RBC agglutination in the cooler parts of the body, such as livedo reticularis, Raynaud disease, acrocyanosis, and, in rare cases, cutaneous necrosis [1]. Exacerbations of hemolysis can be triggered by infection, trauma, or surgery [1,2]. As a result, this condition presents unique considerations during the perioperative period, namely, due to the risk of hypothermia in surgical patients in the setting of impaired thermoregulation.

Impairments in thermoregulatory control are seen in both general and regional anesthesia, resulting in hypothermia [3]. Hypothermia in surgical patients undergoing general anesthesia represents a failure of thermoregulatory defenses primarily due to the redistribution of body heat from the core to the periphery. This redistribution typically results in a decrease in core temperature of 0.5 to 1.5°C [3]. Hypothermia by this same mechanism is also seen in epidural and spinal anesthesia with similar frequency and severity. Additionally, neuraxial anesthesia inhibits the patient’s sensation of cold, which can mask hypothermia if monitoring is not implemented. Other thermoregulatory mechanisms, namely, shivering and vasoconstriction, are impaired in both general, epidural, and spinal anesthesia [3]. Common prevention and management strategies exist to prevent the development of hypothermia in surgical patients. However, despite the similarities in thermoregulatory inhibition between general and neuraxial anesthesia, a paucity of literature exists regarding the perioperative management of patients with cold agglutinin disease, compromising the selection between general or neuraxial anesthesia for lower extremity total joint arthroplasty. 

## Case presentation

An 83-year-old female with a longstanding history of idiopathic cold agglutinin disease presented in August of 2024 for a primary robotic-assisted left total knee arthroplasty (TKA) in the setting of osteoarthritis. Her additional medical comorbidities included hypertension and chronic hepatitis B infection with positive anti-hepatitis B core antibody. Notably, she had episodic hemolytic anemia in the past treated with intravenous immunoglobulin (IVIG), glucocorticoids, and rituximab. Her last cold agglutinin antibody titers, drawn in 2021, were elevated at 1:4096 (normal < 1:32). One year prior to TKA, the patient was hospitalized with *Escherichia coli* bacteremia. During that hospitalization, she experienced a critical episode of hemolytic anemia resulting in several red blood cell (RBC) transfusions (hemoglobin nadir of 3.9 g/dL). After discharge, the patient’s hemoglobin remained stable, and she continued to follow up regularly with her hematologist. Labs obtained one month prior to surgery revealed a stable anemia and reticulocytosis within her baseline with a hemoglobin of 8.8 g/dL and reticulocytes of 6.1%. On the morning of surgery, the patient’s hemoglobin was 8.7 g/dL, near her baseline of 8-10 g/dL.

The patient underwent a primary, robotic-assisted left TKA with the use of a thigh tourniquet under spinal anesthesia with 3.2 mL of 2% mepivacaine within a community hospital setting. Intraoperative sedation was maintained with a propofol infusion. Throughout the patient’s perioperative course, significant efforts were made to avoid hypothermia-induced acute hemolysis through ambient and forced air heating methods. In the preoperative holding area, the patient was pre-warmed with forced air at 42°C (3M™ Bair Huggar™ 3M Inc, Saint Paul, USA). During seated spinal placement, warm blankets were draped across her shoulders and around her head. Her warming gown was run continuously with forced air at 42°C (3M™ Bair Huggar™). An underbody forced-air warmer (3M™ Bair Huggar™) was placed on the operating table and used for the spinal procedure and throughout the operation. Additionally, the chlorhexidine-alcohol applicator was warmed prior to skin preparation and the local anesthetic solution was warmed within a sterile gloved hand prior to administration.

**Figure 1:**
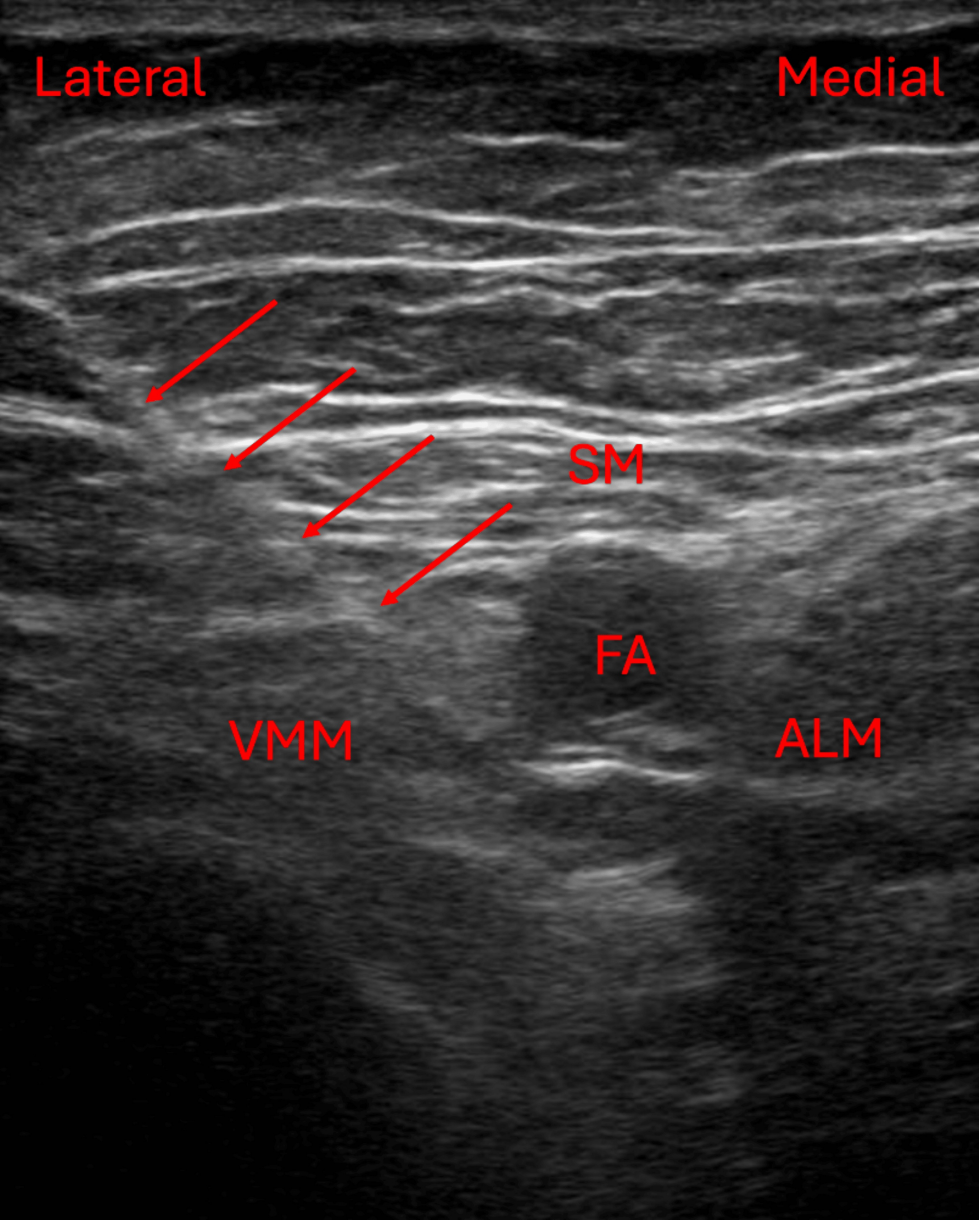
Ultrasound image of this patient's adductor canal block including the needle trajectory (arrows) with the tip of the needle positioned just lateral to the femoral artery within the adductor canal. The femoral artery (FA) courses through the adductor canal, which is bordered by the sartorius muscle (SM) anteriorly, the vastus medialis muscle (VMM) anterolaterally, and the adductor longus muscle (ALM) posteromedially.

After successful spinal placement within 5 minutes, the patient was immediately returned to the supine position upon the underbody forced air warmer with the addition of an over-body forced air warmer shortly thereafter. Additionally, the patient’s head was wrapped in a warmed blanket. Throughout the case, intravenous fluids were warmed to 41°C utilizing a fluid warmer (3M™ Ranger™; 3M Inc, Saint Paul, USA) and the ambient operating room temperature was maintained at 23°C. The patient’s temperature was monitored with a skin temperature probe in the axilla of the left arm which was tucked and secured across the patient's chest. The average temperature recorded throughout the case was approximately 35.9-36.6°C. The estimated blood loss for the case was 100 mL. The total operating room time was 95 minutes. Postoperatively, the patient received a rescue single-injection adductor canal block under ultrasound guidance (Figure 1) with 15 mL of 0.5% bupivacaine with 1:200,000 epinephrine utilizing the same aseptic preparation and local anesthetic solution warming measures as described for the spinal. 

The patient’s perioperative course was uncomplicated, without any signs or symptoms of acute hemolysis. Notably, she was discharged home the following day. During her hematology follow-up 1 month after surgery, laboratory studies revealed a stable hemoglobin at 8.2 g/dL and reticulocytes of 7.8%. These values fell within her established baseline. The increase in her reticulocytes was felt to be due to increased RBC breakdown related to the stress of surgery. Reassuringly, the patient remained asymptomatic with no excessive fatigue. She was restarted on folic acid 1 mg daily with scheduled follow-up and repeat labs two months later. 

## Discussion

**Figure 2:**
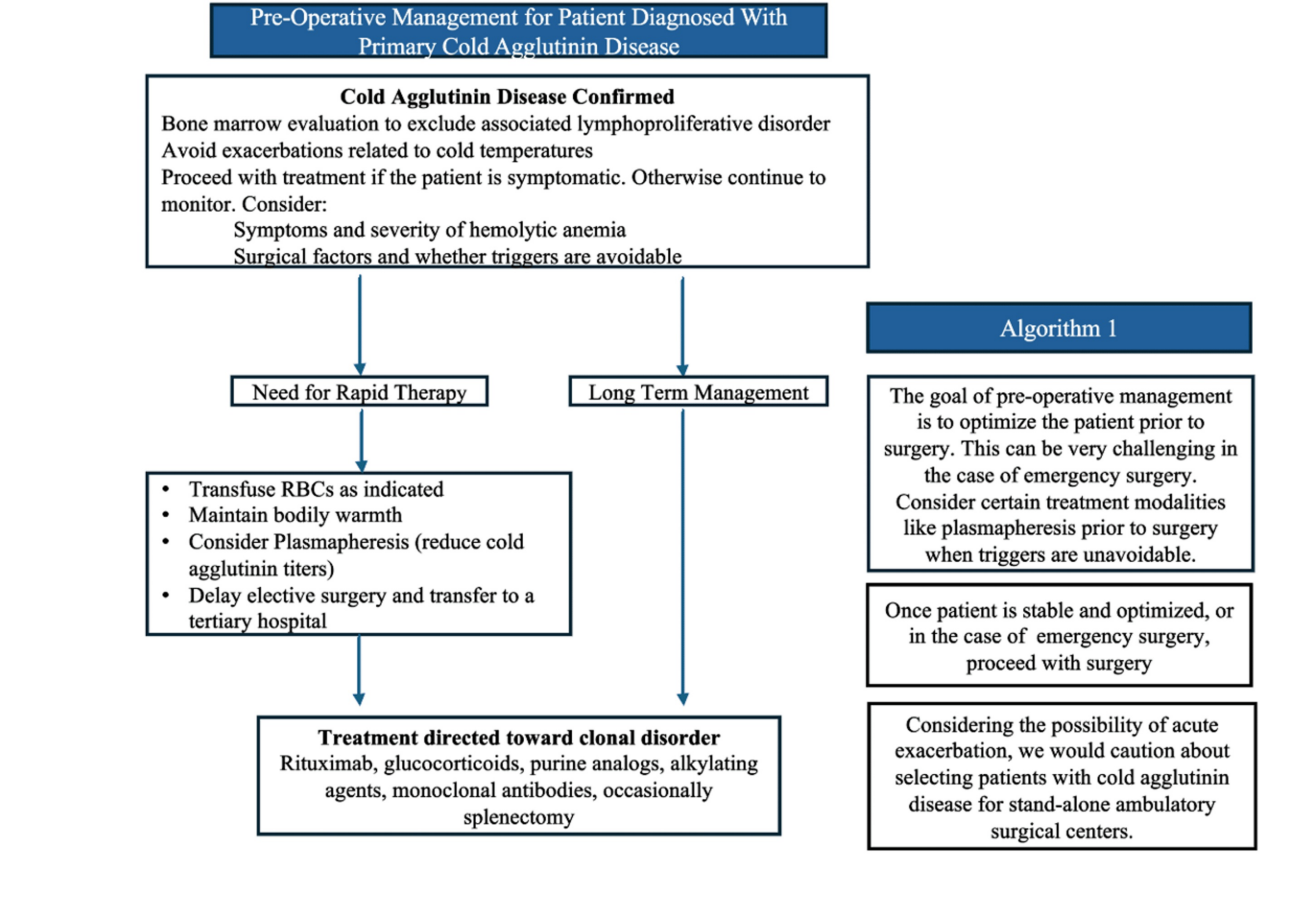
Algorithm 1 depicting a suggested approach for pre-operative optimization for patients with cold agglutinin disease. Figure Credits: This figure is an original figure created by the authors of this case report utilizing PowerPoint (Microsoft Corporation, Redmond, USA)

**Figure 3:**
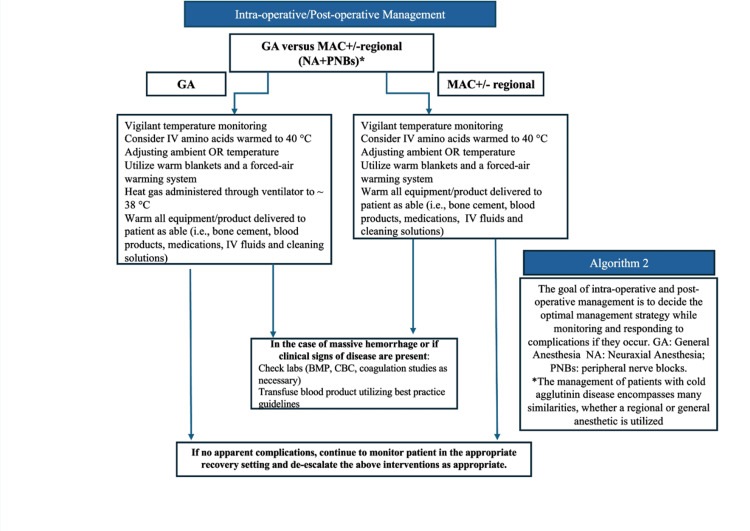
Algorithm 2 depicting a suggested temperature management strategy for patients with cold agglutinin disease undergoing general anesthesia, monitored anesthesia care (MAC), and regional anesthesia. GA: general anesthesia; MAC: monitored anesthesia care; BMP: basic metabolic panel; CBC: complete blood count Figure Credits: This figure is an original figure created by the authors of this case report utilizing PowerPoint.

This report highlights the perioperative considerations for patients with cold agglutinin disease undergoing elective lower extremity orthopedic surgery. Regardless of anesthetic selection, the mainstay of management for this patient population includes preoperative optimization (Figure 2) and perioperative maintenance of normothermia (Figure 3). While core temperature monitoring within the pulmonary artery, distal esophagus, and nasopharynx provides the best estimate of body core temperature [3], monitoring at these sites is not always feasible or practical, especially under neuraxial or regional anesthesia. In our case, we elected not to utilize a nasopharyngeal temperature probe due to the possible discomfort this can cause in a patient not under general anesthesia. Additionally, due to the anticipated short operative time and the limitations of bladder temperature reliability based on urine output [3], a Foley catheter was not placed for temperature monitoring purposes. After correlating with oral temperature values preoperatively, we utilized an axillary temperature skin probe for intraoperative temperature monitoring, which provides a reasonable estimate of core temperature when positioned appropriately near the axillary artery with the patient's arm at the side [3]. The axillary temperature remained stable, and the patient did not show any signs or symptoms of acute hemolysis intraoperatively or postoperatively. 

In addition to aggressive warming techniques intraoperatively, preoperative plasma exchange can be considered for patients who have clinical or laboratory evidence of acute hemolysis, or in patients with high antibody titers. However, this may not be indicated in patients who are stable without acute hemolysis [1,4,5]. Preoperative warming with forced air can effectively reduce the risk of intraoperative hypothermia [6]; it was utilized for our patient. Additionally, warmed amino acid infusions have been utilized as an intraoperative hypothermia prevention strategy due to enhanced thermogenic effects [7]. However, these solutions may not be available in smaller community hospitals or ambulatory surgery centers and were not available for this patient on the day of surgery. Other therapies that have been utilized for the management of cold agglutinin disease include rituximab, cyclophosphamide, chlorambucil, glucocorticoids, intravenous immunoglobulin, and splenectomy [5].

Antibody titer levels do not always correlate with disease activity because hemolysis occurs with levels as low as <1:32, although titers greater than 1:512 are generally considered clinically significant [1]. Given that this patient’s antibody titers were not routinely monitored by her hematologist, and based on her lack of signs or symptoms of an acute exacerbation of her cold agglutinin disease, we did not see an indication to check antibody titers perioperatively. Importantly, antibody titer monitoring would not have been possible within this community hospital setting. When evaluating patients with cold agglutinin disease preoperatively, the presence of any key features of disease exacerbation including livedo reticularis, Raynaud phenomenon, or acrocyanosis [1] would warrant delaying elective surgery, additional laboratory workup, hematology consultation, and potentially transfer to a tertiary care center with hematology services and serologic monitoring capabilities. Key laboratory features, if obtained, include worsening anemia, hyperbilirubinemia, and elevated lactate dehydrogenase [1].

Several publications describe the management of this patient population under general anesthesia including laparoscopic surgery [2], radial cystectomies [4], cardiopulmonary bypass [5], total hip arthroplasty [8], open radial distal gastrectomy [9], and deep hypothermic circulatory arrest [10]. Additionally, the management of obstetric patients with cold agglutinin disease undergoing cesarean delivery has been reported [11]. However, this case report is the first to detail perioperative management of idiopathic cold agglutinin disease in a non-obstetric patient undergoing spinal anesthesia. Advantages of selecting spinal anesthesia include avoidance of intubation, mechanical ventilation, and the unique warming considerations when these techniques are required.

Treatment of cold agglutin disease in cases of non-elective surgery, or in patients presenting with severe, symptomatic hemolytic anemia may differ and may require an alternative strategy (e.g., plasmapheresis) (Figure 2) [4,7,8]. Furthermore, the patient's surgery was offered within a community hospital setting. However, considering the possibility of acute exacerbation, we would exercise caution with selecting patients with cold agglutinin disease for ambulatory settings without immediate access to tertiary care, such as within stand-alone ambulatory surgical centers. The mainstay of management includes aggressive warming techniques, which should include warmed intravenous fluids, warmed blankets, increasing the ambient operating room temperature, and forced air warming methods whenever and wherever possible (Figure 3).

## Conclusions

This report demonstrates the use of spinal anesthesia in a patient with stable idiopathic cold agglutinin disease undergoing orthopedic surgery in a community hospital setting. While patients undergoing spinal anesthesia are at a similar risk of hypothermia, general anesthesia should not be considered the default. Spinal anesthesia can be employed with aggressive warming techniques, continuous non-invasive temperature monitoring, and within an ambulatory setting with immediate access to escalating care services if needed. Additionally, this case highlights the importance of pre-operative optimization of patients with cold agglutinin disease, with special attention paid to disease severity and the risk of acute exacerbation with worsening hemolysis prior to proceeding with elective surgery.
